# Small Molecule Drugs Targeting Non-Coding RNAs as Treatments for Alzheimer’s Disease and Related Dementias

**DOI:** 10.3390/genes12122005

**Published:** 2021-12-17

**Authors:** Lien D. Nguyen, Rachel K. Chau, Anna M. Krichevsky

**Affiliations:** Department of Neurology, Brigham and Women’s Hospital, Harvard Medical School, Boston, MA 02115, USA; lnguyen53@bwh.harvard.edu (L.D.N.); rachelchau@college.harvard.edu (R.K.C.)

**Keywords:** non-coding RNA, Alzheimer’s disease and related dementias, small molecules, drug discovery, high-throughput screens, miRNA, lncRNA, circRNA

## Abstract

Despite the enormous burden of Alzheimer’s disease and related dementias (ADRD) on patients, caregivers, and society, only a few treatments with limited efficacy are currently available. While drug development conventionally focuses on disease-associated proteins, RNA has recently been shown to be druggable for therapeutic purposes as well. Approximately 70% of the human genome is transcribed into non-protein-coding RNAs (ncRNAs) such as microRNAs, long ncRNAs, and circular RNAs, which can adopt diverse structures and cellular functions. Many ncRNAs are specifically enriched in the central nervous system, and their dysregulation is implicated in ADRD pathogenesis, making them attractive therapeutic targets. In this review, we first detail why targeting ncRNAs with small molecules is a promising therapeutic strategy for ADRD. We then outline the process from discovery to validation of small molecules targeting ncRNAs in preclinical studies, with special emphasis on primary high-throughput screens for identifying lead compounds. Screening strategies for specific ncRNAs will also be included as examples. Key challenges—including selecting appropriate ncRNA targets, lack of specificity of small molecules, and general low success rate of neurological drugs and how they may be overcome—will be discussed throughout the review.

## 1. Introduction

### 1.1. ncRNAs as Novel Therapeutic Targets for Treating ADRD

Dementia, the progressive decline in memory and cognitive function, is a major public health problem that affects 40 to 50 million people worldwide [1]. Alzheimer’s disease (AD) is the most common dementia diagnosis and is characterized by the accumulation of Aβ plaques and tau neurofibrillary tangles. In addition to classic AD pathology, a majority of dementia cases also exhibit distinct histologic changes and are further classified as frontotemporal dementia (FTD), Lewy body dementia (LBD), vascular contributions to cognitive impairment and dementia (VCID), and multiple etiology dementias (MED) [2]. Collectively known as Alzheimer’s disease and related dementias (ADRD), they exert devastating burdens on patients, caregivers, and society. Yet, there is currently no effective, disease-modifying treatment for ADRD. The five drugs approved for treating ADRD by the US Food and Drug Administration (FDA), including donepezil, galantamine, rivastigmine (cholinesterase inhibitors), memantine (N-methyl-D-aspartate receptor antagonist), and Aducanumab (Aβ monoclonal antibody), only have modest clinical benefits [3]. Discovering new, more effective therapies for ADRD will, undoubtedly, require innovation on many fronts.

RNAs have been extensively studied for their roles in protein biogenesis. Messenger RNAs (mRNAs) are the intermediary codes between DNA and proteins. Other housekeeping RNAs, including ribosomal RNAs (rRNAs), transfer RNAs (tRNAs), small nucleolar RNAs (snoRNAs), and small nuclear RNAs (snRNAs), participate in modifying and translating mRNAs into proteins. However, recent advances in RNA capturing, sequencing, and annotation have revealed new classes of regulatory non-coding RNAs (ncRNAs) that further enrich the complexity of gene regulations. Whereas 1.5~2% of the human genome is transcribed into mRNAs, ~70% of the genome is pervasively transcribed into ncRNAs [4]. These ncRNAs are broadly divided into small ncRNAs (sncRNAs, <200 nucleotides), including microRNAs (miRNAs) and Piwi-interacting RNAs (piRNAs), and long ncRNAs (lncRNAs, >200 nucleotides), including linear and circular lncRNAs (circRNAs). Table 1 includes some classes of ncRNAs relevant to this review. Physiologically, and particularly in the central nervous system (CNS), ncRNAs play diverse and critical roles in cell division, migration, metabolism, neuronal plasticity, and cell death, through regulating genome folding, gene modifications, transcription, protein synthesis, and scaffolding of protein complexes (for review, see [5]). Notably, many ncRNAs are highly enriched in specific brain regions or cell types [6,7] and are only found in primates and humans [8], supporting the hypothesis that ncRNAs accelerate primate brain evolution and the acquisition of higher-order cognition [9]. Correspondingly, the dysregulation of various ncRNAs contributes to neurodegenerative diseases (for review, see [10]). As such, ncRNAs constitute a large, attractive, and still underexplored pool of possible therapeutic targets for treating ADRD.

### 1.2. Small Molecule Drugs for Modulation of ncRNAs

Extensive work has gone into developing synthetic oligonucleotides to target mRNAs and ncRNAs, including antisense oligonucleotides (ASOs), small interfering RNAs (siRNAs), miRNA mimics and inhibitors, and others (for review, see [11]). However, major hurdles concerning immunogenicity, specificity, and delivery persist [11]. Particularly, delivery of oligonucleotides to tissues other than the liver remains poor, and the brain is further protected by the blood-brain barrier (BBB), which blocks the entry of many compounds [12]. Alternatively, gene therapy utilizing viral vectors can supplement, knockdown, or edit ncRNAs [13]. While potentially allowing for more durable ncRNA modulation, gene therapy also comes with major challenges regarding immunogenicity, delivery, transgene expression in off-target cell populations, and off-target genome editing [13].

In parallel to advancing oligonucleotide and gene therapies for the CNS [13,14,15], small molecules comprise another approach to target ncRNAs. Small molecules typically have low molecular weights (usually <1 kDa) and are administered orally for distribution throughout the body. Their small size, negligible charge, and hydrophobicity allow them to pass the cell membrane and bind to intracellular targets to exert systemic effects [16]. For example, ibuprofen, a non-steroidal anti-inflammatory drug, can readily cross the BBB [17]. In addition to better, “drug-like”, pharmacokinetics and pharmacodynamics (PK/PD), small molecules are typically easier and cheaper to synthesize and modify than synthetic oligonucleotides and viral vectors. Furthermore, as many small molecules are already on the market or in clinical trials with known safety and toxicity profiles, those with newly discovered ncRNA-targeting properties can be repurposed, therefore saving critical time and costs [18].

While targeting disease-relevant proteins with small molecules has long been an integral part of drug discovery, targeting RNAs, and specifically ncRNAs, is an emerging field, with standard rules remaining to be established [4]. Compared to proteins, RNAs have limited chemical diversity, are highly negatively charged, and possess few hydrophobic pockets for conventional drug binding [4,19]. Yet, RNAs can fold into secondary, tertiary, and quaternary structures, with internal loops, bulges, hairpins, and triple helices providing potential binding sites for small molecules [19]. For example, bacterial riboswitches are naturally-occurring RNA regulatory segments that bind metabolites to regulate transcription [19]. In 2020, risdiplam, a small molecule, was the first direct RNA-splicing modifier approved by the FDA for treating spinal muscular atrophy (SMA) [19,20]. Risdiplam directly binds the survival of the motor neuron 2 (*SMN2*) mRNA-spliceosome complex to facilitate exon 7 inclusion and increase SMN protein expression. Small molecules binding to ncRNAs has also been identified as a potential treatment for bacterial infections and cancer [21,22,23,24]. Furthermore, small molecules may regulate ncRNAs without direct binding, for example, by influencing their biogenesis or protein partners as described in Section 2.

In the next section, we will discuss several high-throughput screens (HTS) for discovering small molecules that target ncRNAs and outline the discovery and validation process and their limitations. Special consideration for targeting the CNS for treating ADRD will also be included as appropriate.

## 2. Discovery and Validation of Small Molecules Targeting ncRNAs

**Figure 1:**
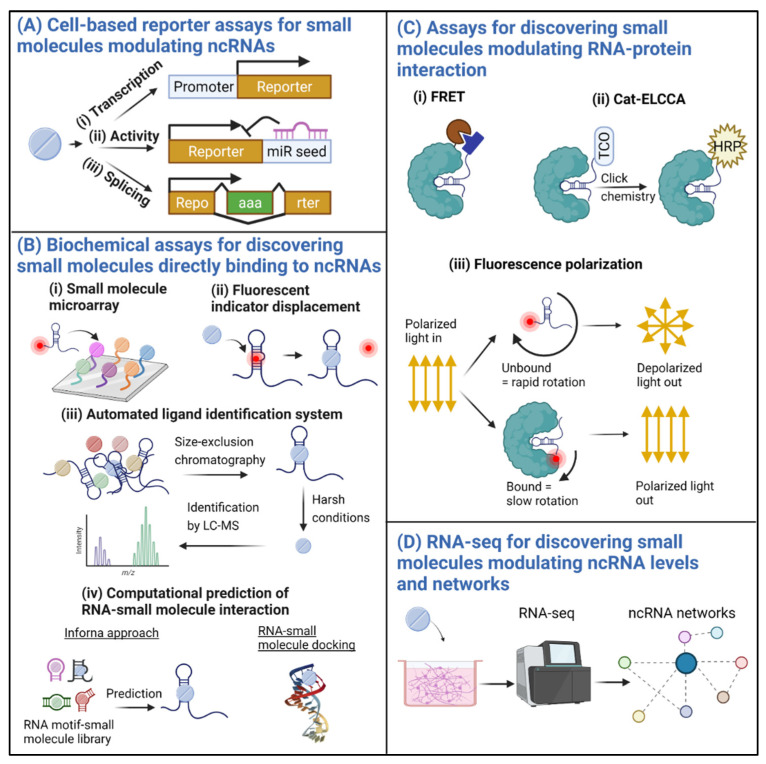
Methods for discovering small molecules modulating the expression level and activity of ncRNAs. (**A**) In the cell-based reporter assays, a fluorescent or luminescent reporter is used to measure ncRNA-expression level or activity: (**i**) if the reporter is under the control of a ncRNA promoter region, small molecules that modulate the reporter may transcriptionally modulate the ncRNA; (**ii**) if the reporter gene is under the control of a region interacting with an ncRNA, small molecules that modulate the reporter may regulate the expression level or activity of the ncRNA; (**iii**) for variants of an ncRNA produced from alternative splicing, small molecules that modulate the in-frame expression of the reporter gene may regulate alternative splicing to favor the production of a specific variant. (**B**) Various biochemical assays can be used to discover small molecules that bind directly to ncRNAs to modulate their configuration and function: (**i**) in the small molecule microarray assay, small molecules are immobilized on glass slides and then incubated with fluorophore-bound ncRNAs. After unbound ncRNAs are washed away, fluorescence signals may indicate binding between a small molecule and the ncRNA; (**ii**) in the fluorescent indicator displacement (FID) assay, the ncRNA is first reversibly attached to a fluorescent indicator. Small molecules that bind to and displace the indicator result in a loss of fluorescent signal; (**iii**) in the automated ligand identification system (ALIS), the ncRNA of interest is first incubated with various small molecules. RNA-small molecule complexes are then separated from unbound RNAs and small molecules by size-exclusion chromatography, followed by treatment under harsh conditions to release the small molecules from the complexes. The small molecules are then identified by liquid chromatography-mass spectrometry (LC-MS); (**iv**) computational methods can also screen millions of small molecules to predict those that are more likely to bind to a particular ncRNA. The Inforna platform utilizes known RNA motif–small molecule interactions to predict compounds for specific RNA secondary structures. RNA structures produced by X-ray crystallography or nuclear magnetic resonance (NMR) can also be used to facilitate small molecule—RNA docking. (**C**) Several assays can also be used to discover small molecules that modulate RNA-protein interaction: (**i**) in the fluorescence resonance energy transfer (FRET) assay, the protein can be tagged with a fluorophore, whereas the ncRNA is tagged with either an activator or a quencher; (**ii**) in the catalytic enzyme-linked click chemistry assay (cat-ELCCA), the protein is immobilized, while the RNA is tagged with a handle such as a 5′-trans-cyclooctene (TCO) that can be converted into horseradish peroxidase (HRP) using click chemistry; (**iii**) in the fluorescence polarization assay, small molecules bound to proteins rotate more slowly than unbound molecules, resulting in more highly polarized light emission. Small molecules that modulate the fluorescent, or luminescent, or polarized light intensity in these assays may also modulate ncRNA-protein interaction. (**D**) High throughput ncRNA-seq can be used to directly identify ncRNAs that are differentially expressed in cellular models treated with small molecules. This approach also enables the construction of ncRNA networks altered by a particular small molecule.

A small molecule may regulate an ncRNA by binding directly to the ncRNA to change its conformation; or by initiating a signaling cascade that alters its biogenesis, splicing, function, or stability; or by interfering with the formation of the ncRNA-protein complex. Methods utilized to discover drugs that target proteins have been repurposed for targeting RNAs with varying degrees of success. So far, most studies have focused on small molecules that target bacterial or viral RNAs or ncRNAs in cancer [20]. Small molecules that bind to and modify the splicing of pre-mRNAs (for example, *SMN2* or microtubule associated protein tau (*MAPT*)) have also been investigated [25,26,27]. However, we are not aware of any study that uses small molecules to target ncRNAs in neurodegenerative diseases. We posit that small molecules that target ADRD-relevant ncRNAs can be discovered using similar methods (Figure 1). However, more so than other diseases, small molecules that target the CNS and potentially require chronic administration need to satisfy additional stringent criteria, including BBB permeability, acceptable general and brain-specific PK/PD, high specificity, and low toxicity.

### 2.1. Selection of Compound Libraries and Cellular or Animal Models

The distinct advantage of small molecules over synthetic oligonucleotides or viral vectors is the wealth of knowledge, the variety of compounds, and the relatively low cost of synthesis and modification. For example, the ZINC15 database lists 230 million purchasable small molecules (as of November 2021), with each compound annotated for its chemical property, biological activity, and commercial availability [28]. The selection of the size and diversity of a screening library relies on various factors, including cost, quality, availability, chemical diversity, assay sensitivity, single or multiple concentrations, and estimated hit rate, as well as specific properties of the ncRNA target [29]. To maximize potential novel discoveries, large libraries (>50,000) of compounds of diverse chemical structures can be utilized. However, it should be noted that existing libraries are still optimized for protein binding, not RNA binding [19]. For example, a recent screen for direct binders using the Merck Diversity Library (~50,000 compounds) has an average hit rate of 0.05% for proteins and 0.01% for RNAs, while the Functionally Annotated Library (~5100 compounds) has an average hit rate of 1.54% for proteins and 0.04% for RNAs [23]. Alternatively, more focused specialty libraries can be utilized, including ZINC15’s substances active in cells (~34,000 compounds), Selleck’s Approved Drug Library (~3000 compounds), Sigma-Aldrich’s Library of Pharmacologically Active Compounds (LOPAC, 1280 compounds), or a recently curated list of BBB-permeable compounds (~5000 compounds, [30]). In addition, libraries of chemical fragments (<300 Da) can be used to detect fragments with mM binding affinity to guide syntheses of higher-affinity compounds [29]. Finally, while computational predictions of small molecule-RNA binding remain to be improved, they can be used to narrow down millions of compounds to a few thousand for experimental validation.

Few drugs discovered through academic preclinical research advance to human clinical testing, and, of those, the vast majority fail at various stages. Between 2010 and 2017, only 7% of new drugs that entered phase I clinical trials eventually made it to the market, with drugs targeting the nervous system having the lowest success rate, at only 3% [31]. Particularly, the success rate for disease-modifying treatments for ADRD is still 0%. Selecting the appropriate cellular and animal models, either in the primary screen or in follow-up validation studies, is critical for reducing the failure rate. For example, most cell-based screens still utilize a single immortalized cell line grown in media designed for rapid growth and on artificial 2D substrates [32]. In contrast, neurons are primarily non-dividing and grow in complex 3D environments with other neurons, astrocytes, oligodendrocytes, and microglia from diverse lineages. As such, more relevant cellular models for screening or validation include primary or induced pluripotent stem cell (iPSC)-derived neuronal and glial cells, organotypic brain slices, and brain organoids [32]. However, such models are also more heterogenous and challenging for screen design, and so far, they are rarely utilized in HTSs. Specific neuronal-like cell lines with neurodifferentiation potentials, such as SH-SY5Y cells, may be considered a compromise between technical feasibility and disease relevance. Subsequently, the most promising small molecules need to be tested in animal models to establish brain PK/PD and efficacy at the molecular, cellular, and behavioral levels. Determining adequate replication and group sizes, potential sex-specific effects, evidence of target engagement, treatment timing, and testing in multiple disease-relevant models is critical for enhancing reproducibility and translatability to clinical studies [33].

### 2.2. Cell-Based Reporter Assays for Discovering Regulators of ncRNAs

Most regulatory ncRNAs, including many miRNAs [34], antisense transcripts (AS), and lncRNAs [35], are transcribed similarly to mRNAs by RNA polymerase II. circRNAs are produced from the processing of pre-mRNAs [35]. Therefore, similar to mRNAs, these ncRNAs are regulated by mechanisms involving promoters, transcription factors, enhancers, and suppressors. International collaborative efforts, including the Encyclopedia of DNA Elements (ENCODE) [36] and the Functional Annotation of the Mammalian Genome (FANTOM) [37] consortia, have extensively annotated regulatory elements of the genomes of various species, allowing for the prediction of regulatory regions of ncRNAs. For example, the promoters of 1357 human and 804 mouse miRNAs were recently annotated [38]. In promoter-driven screens (Figure 1A(i)), the promoter of an ncRNA of interest can be cloned into plasmids to control the expression level of a fluorescent or luminescent reporter. Upon the addition of small molecules, the intensity of the reporter is used as a readout for promoter activity—with lower intensity indicating reduced activity and vice versa. A recent screen of 52,041 compounds identified 739 compounds that decreased huntingtin (*HTT*) promoter-driven luciferase activity and 52 that increased *HTT-AS* promoter-driven luciferase activity in HEK293 cells [39]. Seventeen of those compounds were shown to reduce HTT protein levels but at concentrations also toxic to cortical-like neurons differentiated from iPSCs from Huntington’s disease patients.

The reporter assay can also be used to measure ncRNA activity by placing the reporter gene under the control of the region interacting with the ncRNA of interest, for example, the 3′ untranslated region containing miRNA binding sites (Figure 1A(ii)). The intensity of the reporter is then used as the readout for ncRNA activity. Such screens have been used to identify small molecules that modify the activity of miR-21 [40,41,42], miR-34a [43], and miR-122 [44] in cancer cells. Alternatively, a reporter assay can be used to screen for RNA splicing modifiers similar to risdiplam. Splicing is important for producing variants of ncRNAs with distinct functions, including variants that gain or lose coding potentials [45]. In particular, the production of circRNAs requires the back-splicing of linear transcripts [35,45]. In the splicing reporter assay, the inclusion or exclusion of an exon results in the in-frame expression of the reporter gene, allowing for the identification of compounds that regulate the splicing process (Figure 1A(iii)). A reporter system was recently used to identify molecular elements critical for back-splicing [46], which may be repurposed to screen for small molecules that promote circRNA expression. Additionally, with the knowledge of the regulatory sequences of ncRNAs, the putative transcription factors that regulate the ncRNAs can be predicted. For example, the TransmiR database lists the possible transcription factors for many miRNAs based on published studies, chromatin immunoprecipitation—RNA-seq (ChIP-seq) data, and predicted binding motifs [47]. Knowing the pathways that regulate these transcription factors, in turn allows for the selection of specific classes of small molecules for smaller-scale, targeted screening.

A possible challenge for cell-based reporter assays is the technical difficulty of obtaining uniform transfection efficiency and expression level of the reporter gene in miniaturized formats, especially for heterogeneous systems such as primary cultures, organotypic slices, and brain organoids. For example, compared to immortalized cell lines, primary neurons have lower transfection efficiency and show reduced survival or viability following transfection [48]. Reporter signals may also be confounded by small molecules that directly inhibit or enhance the reporters, for example, small molecules that inhibit luciferase [49]. In addition, the mechanism of actions (MoAs) of positive hits needs to be determined. Ideally, the MoAs of positive hits should be novel or related to disease-relevant pathways [50]. However, the hits may also have non-specific MoAs or MoAs resulting in toxic effects downstream.

### 2.3. Biochemical Assays for Discovering Direct Binders of ncRNAs

Much work has been invested in identifying direct binders of ncRNAs, inspired by decades of research for small molecules that bind proteins. Recent biochemical assays and computational predictions have successfully identified small molecules that directly bind ncRNAs to modify their life cycles and functions. Depending on the screening methods, the compound libraries, the target ncRNAs, and the selection criteria, between 0.01~9% of compounds tested were identified as positive hits from the primary HTSs [20,23].

In the small molecule microarray assay (Figure 1B(i)), small molecules are spatially arrayed and covalently bound to glass slides and then incubated with the fluorescently labeled RNA of interest [51]. After washing, glass slides are imaged with a fluorescence scanner to identify positive hits, for example, compounds that bind to and modulate riboswitch activity [52]. In the fluorescent indicator displacement (FID) assay (Figure 1B(ii)) [53], the RNA target is first incubated with a fluorescent indicator that reversibly binds RNA, followed by incubation with a library of small molecules. Compounds that bind to the RNA and displace the fluorescent indicator result in the loss of fluorescence signal. However, compounds that bind the RNA without displacing the indicator may not be detected as positive hits. FID using SYBR Green II as the indicator was employed to compare the binding efficiency of compounds that bind the lncRNA metastasis-associated lung adenocarcinoma transcript 1 (*MALAT1*) triple helix motif [21]. In the automated ligand identification system (ALIS) (Figure 1B(iii)), [23], RNA-small molecule complexes are first separated from unbound components by size-exclusion chromatography. Small molecules are then released from the complexes and identified by liquid chromatography-mass spectrometry (LC-MS). A recent screen of 42 RNAs of diverse classes against 50,000 small molecules identified direct binders for 40 RNAs, with an average hit rate of 0.01% per RNA [23]. Notably, 30 RNAs had one or more selective binder that did not bind any other RNA in the screen.

The discovery of direct RNA-small molecule interactions has also provided the basis for bioinformatics tools that facilitate in silico predictions of novel small molecules that bind to specific RNAs sequences (Figure 1B(iv)). For example, the Inforna platform incorporates known RNA motif–small molecule interaction to predict lead compounds for RNA secondary structures [54]. Inforna was used to identify lead compounds that target miR-96, which were optimized into Targaprimir-96, which binds pri-miR-96 to inhibit its processing into the oncogenic mature form [24]. Deeper analyses of known RNA-small molecule interactions further suggest that these compounds tend to contain aromatic amine-containing heterocycles or amidine-like motifs conducive for stacking and hydrogen-bonding interactions with their RNA targets [23,55]. Additionally, protein crystal structures obtained from X-ray diffraction and deposited into the Protein Data Bank (PDB) allow for computational docking of millions of small molecules to facilitate structure-based drug discovery [56]. The PDB also contains crystal structures of RNA-small molecule complexes (PDB ID: 3C44, HIV-1 extended duplex RNA bound to paromomycin HIV-1 [57]; PDB ID: 4QLM, ydaO riboswitch bound to c-di-AMP [58]). Nevertheless, compared to proteins, RNAs are often more dynamic with more disordered regions, making them harder to crystalize [19]. For short and disordered RNAs, nuclear magnetic resonance (NMR) spectroscopy utilizes magnetic fields to determine their structures and possible conformational changes induced upon binding to small molecules (PDB ID: 5UZT, pre-miR21 structure [59]). The increase in the number of solved and deposited RNA-small molecule complexes is expected to improve in silico predictions. Both X-ray diffraction and NMR can also be used to validate direct RNA-small molecule interaction of positive hits from the primary screen. Other validation methods include surface plasmon resonance (SPR), isothermal titration calorimetry (ITC), cryoEM, and chemical probing (for review, see [19]). In reverse, Chemical Cross-Linking and Isolation by Pull-down to Map Small Molecule-RNA Binding Sites (Chem-CLIP-Map-Seq) can be used to confirm the specificity of RNA-small molecule interaction [60]. Briefly, a small molecule conjugated to beads is crosslinked to its RNA targets, which are then identified through RNA-seq, therefore providing information about the specificity of the interaction between the small molecule and the RNA of interest.

While selective RNA binders are expected to act more specifically than indirect regulators, a limitation is that the binding of small molecules to ncRNAs almost always inhibits their functions, either by blocking their maturation [24], inducing their degradation by endogenous RNases, or interfering with their structure or activity [61] (for review, see [62]). On the other hand, we have not found studies of small molecules that stabilize and enhance the functions of ncRNAs through direct binding, necessitating further studies. In addition, as most regulatory ncRNAs are expressed at much lower levels than rRNA, tRNAs, and snRNAs [63], direct small molecule binders may be sequestered away by the more abundant RNA species even if the latter has a much lower binding affinity, therefore requiring higher concentrations for the desired effect.

### 2.4. Assays for Discovering Small Molecules Modulating RNA–Protein Interaction

The life cycle and functions of ncRNAs are intertwined with their protein partners. For example, miRNA maturation and export involves Drosha, DiGeorge critical region 8 (DGCR8), Exportin 5 (Exp5), Dicer, and the transactivating response RNA-binding protein (TRBP) [34]. miRNAs’ function then requires loading the guide miRNA strand into the RNA-induced silencing complex (RISC) composed of Argonaute. Other proteins bind to and regulate more specific clusters of miRNAs (see [34] for review). For example, Lin28 binds and negatively regulates the let-7 family, miR-9, miR-107, and miR-143. The lncRNA *HOX* antisense intergenic RNA (*HOTAIR*) recruits proteins in the polycomb repressive complex 2 (PRC2) to catalyze H3K27me3 markers to modulate transcription. Interfering with the interaction between ncRNAs and their RNA binding proteins (RBPs) may also be a therapeutic strategy to modulate the abundance and functions of ncRNAs.

Various fluorescence- or chemiluminescent-based methods can be used to detect small molecules that interfere with RNA–RBP interaction [64]. In the fluorescence resonance energy transfer (FRET) assay (Figure 1C(i)), the RBP can be tagged with a fluorophore, and the RNA of interest can be tagged with either a quencher or an activator. Second, in the catalytic enzyme-linked click chemistry assay (cat-ELCCA) (Figure 1C(ii)), the protein is immobilized, whereas the RNA is tagged with a handle. Using click chemistry, the RNA is then conjugated with horseradish peroxidase (HRP), allowing for chemiluminescent detection upon the addition of an HRP substrate. Third, in the fluorescence polarization (FP) assay (Figure 1C(iii)), the RNA is tagged with a fluorophore that rotates rapidly in its unbound state. The binding of RNA to a larger RBP reduces the rotation rate, resulting in increased polarized light emission. In these assays, small molecules that change fluorescent or chemiluminescent intensity, or polarized light emission, are candidate modulators of RNA–RBP interaction. FRET [65], FP [66], and cat-ELCCA [67] assays were used successfully to identify small molecules that disrupt Lin28/let-7 interaction.

Small molecules that interfere with RNA-protein binding can do so either by binding to the RNA or the protein. Notably, the three studies that discovered small molecules disrupting Lin28/let-7 interaction also demonstrated that these compounds bound to Lin28, not let-7 [65,66,67]. However, small molecules that bind to proteins with ubiquitous functions may act non-specifically. For example, small molecules that bind Dicer [68] or Argonaute [68,69] likely affect general miRNA activity, leading to non-specific downstream effects.

### 2.5. RNA-seq for Discovering Small Molecules That Modulate ncRNA Levels and Networks

The various methods described above focus on examining a single ncRNA or a small group of closely related ncRNAs. Applying RNA-seq to discover drugs targeting ncRNAs promises comprehensive, unbiased data on many ncRNAs. Furthermore, sequencing does not rely on artificial reporters and can be more easily used for neuronal cells that are difficult to transfect with reporter constructs. However, besides cost concerns, compared to mRNA-seq, ncRNA-seq, particularly for lncRNA, remains more challenging because of their low abundance, lack of evolutionary conservation, and incomplete genomic annotations [70]. While methods are being developed for more accurate transcriptomic studies of sncRNAs, lncRNAs, and circRNAs [71], and a mouse tissue atlas of sncRNAs was recently published [72], we are not aware of any ncRNA-seq-based efforts for small molecule discovery specifically. Nevertheless, recent advances in next-generation sequencing have made it cheaper and faster to sequence complete sets of RNA transcripts in HTS drug-discovery platforms. For example, the Digital RNA with pertUrbation of Genes (DRUG-seq) platform was used to profile 433 compounds across 8 doses at ~$2–4 per sample [73]. More recently, targeted organoid sequencing (TORNADO-seq) was used to profile the effects of 320 compounds on 206 genes in wildtype and tumor intestinal organoids at $5 per sample [74]. These new developments suggest that ncRNA-seq for small molecule drug discovery will become more feasible in the near future.

In addition, compared to synthetic oligonucleotides, which can recognize specific target sequences through complementary base pairing, small molecules are more likely to act non-specifically. Beyond the ncRNA of interest, small molecules probably perturb other mRNAs and ncRNAs, potentially leading to adverse effects. The adverse effects of some drugs, for example, cognitive impairment following chemotherapy [75], may be outweighed by their clinical benefits or may be addressed by the post-hoc development of combination therapy counteracting the side effects. As such, knowing how small molecules affect pathways other than the intended targets can help predict and alleviate adverse effects before entering human clinical testing. Notably, microarray gene expression data was used to predict adverse drug effects, therefore informing go/no-go decisions in the early stage of drug discovery [76], suggesting that RNA-seq data can not only be used for the primary HTSs but also for subsequent functional and specificity validation assays.

### 2.6. Validation and Optimization of Candidate Small Molecules from Primary HTSs

**Figure 2:**
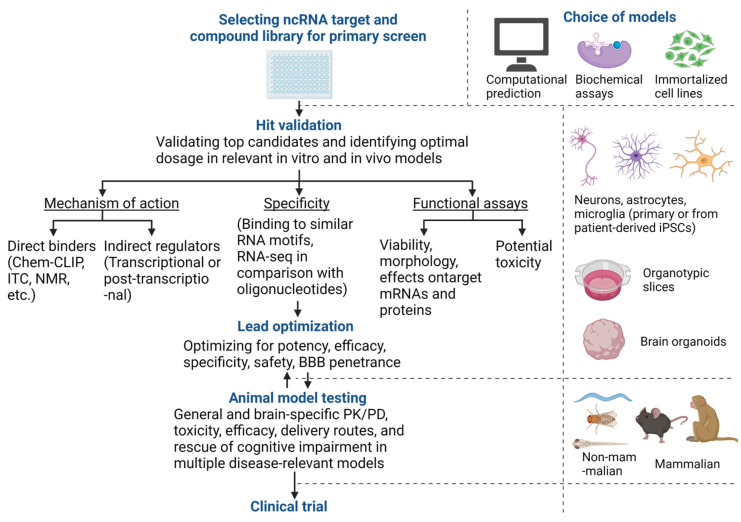
Preclinical discovery workflow for therapeutic small molecules targeting ncRNAs. This figure briefly outlines the workflow for discovering and validating small molecules targeting ncRNAs, with the recommended cellular and animal models at various steps on the right. The workflow starts with selecting an ADRD-relevant ncRNA target, a compound library, and a screening method. The size of the compound library, from a few hundred to millions of small molecules, will depend on various factors, including cost and estimated hit rate. For convenience and cost concerns, the primary HTS can be performed computationally, in biochemical assays with synthetic ncRNAs and small molecules, in immortalized cell lines, or, if possible, in ADRD-relevant cellular models. Once several candidates of small molecules have been identified from the primary screen, they should be validated in ADRD-relevant cellular models, including primary cells from animal models, cells differentiated from patient-derived iPSCs, organotypic brain slices, and brain organoids. An optimal dose that maximally modulates the ncRNA target should first be established. Subsequently, the mechanism of action, the specificity of action, and the functional efficacy and potential toxicity of each candidate small molecule can be investigated. Small molecules that specifically modulate the target ncRNA and its downstream targets and show neuroprotective effects with minimal toxicity can be further optimized with medicinal chemistry for improved potency, efficacy, safety, and BBB penetrance. Next, these improved small molecules can be tested for evidence of efficacy at the cellular and behavioral levels in multiple animal models of ADRD, followed by further chemistry optimization as needed. Finally, a small molecule with specific ncRNA target engagement, satisfactory brain PK/PD, good safety, and evidence of efficacy in animal models may be able to progress to human clinical trials. Abbreviations: Chem-Clip: Chemical Cross-Linking, and Isolation by Pull-down; ITC: Isothermal titration calorimetry; NMR: nuclear magnetic resonance; BBB: blood–brain barrier; PK/PD: pharmacokinetics and pharmacodynamics; iPSCs: induced pluripotent stem cells.

Figure 2 outlines the process from discovering small molecule candidates to validation assays, then to obtaining data that may support progress to human clinical trials. Ideally, the initial screen of several thousands of small molecules identifies a few dozen to hundreds of small molecules as promising hits. Compounds that generate false positives such as pan-assay interference (PAIN) compounds [77], non-specific DNA/ RNA intercalators [20], and luciferase inhibitors [78] should be considered critically and excluded using orthogonal assays at the early stage of the pipeline. Subsequently, the remaining candidate compounds can be resynthesized and validated through a battery of functional assays, further optimized, and then tested in animal models to obtain preclinical data.

Briefly, for the validation process, the expected MoA between a small molecule and an ADRD-related ncRNA should first be verified. For direct binders, the binding site between a small molecule and an ncRNA can be determined by X-ray crystallography, cryoEM, or NMR, while the stoichiometry and thermodynamic properties of the interaction can be determined using ITC and SPR. Chem-CLIP-Map-Seq can also be used to determine if the small molecule may interact with other structurally similar RNAs. For non-binders, the mechanism of ncRNA modulation should be determined, for example, whether the small molecule transcriptionally modulates the ncRNA or whether it promotes the processing of the ncRNA. Next, to determine the specificity of the small molecule, RNA-seq comparing cellular models treated with the small molecule or with relevant oligonucleotides should be performed, as oligonucleotides are often more specific than small molecules. Most importantly, the small molecule should show desirable functional effects, for example, decreasing amyloid aggregates or improving neuronal survival with minimal toxicity in relevant ADRD cellular models.

Small molecules identified in the primary screen can be further optimized with medicinal chemistry (for example, through structure–activity relationship (SAR) analysis) to improve potency, efficacy, and specificity and to minimize toxicity. Subsequently, small molecules can then be tested in several animal models to establish evidence of CNS entry and general and brain-specific PK/PD. Inefficient drug delivery to the CNS is a major challenge for treating ADRD, as well as glioma, stroke, and traumatic brain injury [79]. Some small, hydrophobic drugs can cross the BBB. For example, risdiplam is taken orally and crosses the BBB to increase functional SMN proteins in the CNS [80]. However, if oral administration does not lead to sufficient levels of small molecules in the CNS, other routes of delivery can be considered, including intravenous infusion, intranasal delivery, intrathecal delivery to the cerebrospinal fluid, intraventricular infusion, and focused ultrasound to transiently disrupt the BBB [79]. For the selected delivery route, maximally tolerated dose, target engagement, and efficacy at the molecular, cellular, and behavioral levels can be established. A small molecule with extensive evidence of safety and efficacy in animal models may then be considered a candidate for advancement into human clinical testing.

## 3. Examples of ADRD-Relevant ncRNAs and Screening Strategies

### 3.1. Selecting ncRNA Targets and Screening Strategies

Similar to selecting a compound library, selecting appropriate ncRNA targets and screening strategies is critical and involves many factors. Generally, for any disease, the ncRNA target should directly contribute to the pathophysiology of a disease, and modification of this ncRNA can reverse or delay the course of the disease without significantly impacting physiological functions [81]. For ADRD specifically, the timing of therapeutic interventions is also important. Interventions can be categorized as primary prevention before the first pathophysiological changes occur, secondary prevention when pathophysiological changes start to accumulate but the patient is cognitively normal, or symptomatic treatment when the patient starts to show mild cognitive impairment and eventually progresses to full-blown ADRD [80]. With primary diagnosis and prevention remaining challenging, secondary prevention may currently be the desirable sweet spot where ADRD can be detected up to 20 years before noticeable and irreversible cognitive decline occurs, though symptomatic treatment is also an essential clinical need. As such, ncRNA targets for secondary prevention are likely to be dysregulated before extensive cell deaths occur. In Table 2, we list various ncRNAs that have been shown to be dysregulated in human ADRD patients, the pathways implicated in ADRD pathology, as well as evidence of therapeutic potential in cellular and animal models. These include various miRNAs that have been extensively studied and validated. Others such as circRNAs, piRNAs, and enhancer RNAs (eRNAs) were discovered more recently and are therefore less well-studied but may emerge as promising candidates with further study.

In addition, the screening strategy selected will depend on the ncRNA of interest. For example, lncRNAs may be more amenable to direct binders than mature miRNAs, which are likely too short to possess well-defined binding pockets. In the following section, we briefly discuss the screening strategies for discovering small molecule drugs, using three relatively well-validated ncRNAs as examples.

### 3.2. Upregulating miR-132, a miRNA Commonly Downregulated in ADRD

We and other labs have shown that miR-132 is a key miRNA downregulated in the early stages of ADRD before significant neuronal death occurs [82,83,84,85,86,87,88,89]. miR-132 overexpression via viral vectors or oligonucleotide mimics exerts neuroprotective effects, delays neurodegeneration, and rescues behavioral deficits in several animal models of ADRD [92,96]. However, targeting miRNAs through a direct small-molecule binder would more likely lead to miR-132 inhibition, as seen for small molecules binding to precursors of other miRNAs [24,61,132]. Since miR-132 has a well-defined promoter region regulated by transcription factors including the cAMP-response element-binding protein (CREB), the brain-derived neurotrophic factor (BDNF), and the RE1-silencing transcription factor (REST) [133], a promoter-driven luciferase reporter assay may identify small molecules that transcriptionally upregulate miR-132. Alternatively, high-throughput miRNA-seq may also be utilized to identify small molecules that upregulate miR-132 either directly or indirectly.

### 3.3. Inhibiting BACE1-AS, a lncRNA Upregulated in ADRD

β-secretase 1 antisense transcript (*BACE1-AS*) is a lncRNA and the antisense transcript of *BACE1* and is upregulated in various brain regions of ADRD patients [111,112]. *BACE1-AS* binds to and stabilizes *BACE1* mRNA, resulting in increased BACE1 protein expression, enhanced APP processing, Aβ1–42 production, and plaque formation. Therefore, reducing *BACE1-AS* expression level or functional activity may alleviate Aβ plaque. Another lncRNA, *MALAT1*, was successfully targeted with direct and selective small molecule binders through small-molecule microarray [22] and FID assays [21]. As such, identifying small molecules that directly bind various regions of *BACE1-AS* may decrease its expression level or inhibit its binding to *BACE1* mRNA to reduce Aβ plaque load.

### 3.4. Modulating the Splicing Efficiency of circRNAs Dysregulated in ADRD

circRNAs are highly stable and usually exhibit tissue- or cell type-specific expression, therefore making them promising therapeutic targets [134]. Cerebellar degeneration-related protein 1 antisense RNA (*CDR1-AS*) is one of the first and most well-studied circRNAs. The *CDR1-AS* sequence contains several miR-7 binding sites to sequester miR-7 away from its targets, therefore inhibiting miR-7 activity [129]. *CDR1-AS* is highly expressed in human and mouse brains [129] and is reported to be downregulated in the hippocampus and temporal cortex of AD patients [126], although a different study reported a small upregulation in AD parietal cortices [127]. Two separate studies also found a downregulation of *circHOMER1* and *circKCNN2* and an upregulation of *circDOCK1* in AD frontal and parietal cortices [127,131]. To identify small molecules that modulate the back-splicing, and hence biogenesis, of circRNAs, the splice sites of these RNAs can be cloned into a plasmid in which the successful back-splicing and circularization of the RNA results in the in-frame expression of a reporter [46]. As such, small molecules that modulate the reporter intensity may also be able to modulate circRNA expression.

## 4. Conclusions and Future Perspectives

Despite intensive research and significant advances in our understanding of ADRD genetics and pathophysiology, a disease-modifying treatment with significant clinical benefits has still not been identified. Directly modulating well-established ADRD targets, including Aβ, PSEN1, PSEN2, BACE1, Tau, and APOE, have yielded limited success [135]. Regulatory ncRNAs, which have only been discovered, annotated, and investigated in the last two decades, represent novel therapeutic targets for treating ADRD. This review describes various methods to identify small molecules that modulate ncRNAs as potential ADRD treatments. Going forward, some key challenges are selecting the most relevant ncRNA targets, the potential off-target effect of small molecules, and the low translatability from basic research to clinical testing for neurological diseases. For the first challenge, more studies into novel, brain-enriched, and specific ncRNAs, their physiological functions, and how they are dysregulated in the early stages of ADRD will provide information on ADRD-relevant targets. Notably, most ADRD-associated genetic variants identified in genome-wide association studies are located in non-coding regions [136], suggesting that some variants maybe transcribed into aberrant ncRNAs that can be targeted therapeutically. For non-specificity, medicinal chemistry can help with optimizing the specificity, safety, and BBB-permeability. Finally, performing screen and validation studies in ADRD-relevant cellular and animal models is important for reproducibility and maximizing translatability. Combining the extensive knowledge of ADRD pathophysiology and small molecules with the emerging field of ncRNA biology will perhaps lead to novel, truly disease-modifying medications for ADRD.

## Figures and Tables

**Table 1:** Different classes of ncRNAs.

**Small non-coding RNAs:** ncRNAs of <200 nucleotides.	
MicroRNAs	ncRNAs of 18–25 nucleotides that facilitate the degradation or inhibit the translation of mRNA targets through imperfect complementary base pairings.
Piwi-interacting RNAs	ncRNAs of 26–31 nucleotides that facilitate the silencing of transposons in germline cells, may also have other functions in somatic cells.
tRNA-derived small RNAs	tRNA-derived fragments of 14–30 nucleotides and tRNA halves of 30–50 nucleotides produced from precursor or mature tRNAs. Emerging evidence suggests that they function as signaling molecules in stress responses and as regulators of gene expression.
**Long non-coding RNAs (lncRNAs):** ncRNAs of >200 nucleotides, with largely elusive functions. They can be further classified by the genomic loci from which they are transcribed.	
Intergenic lncRNAs	lncRNAs transcribed from regions not overlapping with protein-coding genes.
Intronic lncRNAs	lncRNAs produced from introns of protein-coding genes.
Sense-overlapping lncRNAs	lncRNAs transcribed from regions overlapping with introns and exons of protein-coding genes.
Bidirectional lncRNAs	lncRNAs transcribed from the same promoters of protein-coding genes, but in the opposite direction.
Antisense lncRNAs	lncRNAs transcribed from the antisense RNA strands of protein-coding genes.
Enhancer RNAs	lncRNAs transcribed from genomic enhancer regions.
Circular RNAs	Closed single-stranded lncRNAs produced by back-splicing, in which the 5′ and 3′ termini of linear RNAs are covalently joined by spliceosome-mediated splicing. Most known circRNAs are transcribed from protein-coding genes.
Pseudogene transcripts	RNAs transcribed from DNA sequences that resemble protein-coding genes but lack the ability to produce functional proteins. These transcripts maybe processed into siRNAs or function as endogenous miRNA sponges.

**Table 2:** Some ncRNAs dysregulated in ADRD with evidence for therapeutic potential.

RNA Species	Evidence of Dysregulation in Human ADRD	Signaling Pathways and Genes Affected	Therapeutic Application and Potential	
			In Cell Lines	In Animal Models
**Small ncRNAs**				
miR-132	Downregulated in AD hippocampus, prefrontal cortex, temporal cortex [82,83,84,85,86,87,88,89]; significantly associated with Braak score in some studies Downregulated in FTD frontal cortex [90,91]	Aβ homeostasis: *ITPKB*, *ERK1/2*, *BACE1* [87] Tau homeostasis: *MAPT*, *CAPN2*, *RBFOX1*, *GSK-3β*, *EP300*, *PP2B*, *ITPKB* [82,87,92,93] Neuronal apoptosis: *PTEN, FOXO3a* [82] Neurogenesis and synaptic plasticity: *p250GAP, MeCP2, BDNF* [94,95,96]	miR-132 viral overexpression or mimics rescued peroxide, glutamate, and Aβ toxicity in primary rodent and human neurons [82,92]	Viral overexpression and mimics rescued hippocampal cell death, tau homeostasis [92,93], hippocampal adult neurogenesis, and behavioral deficits in various AD mouse models [93,96]
miR-107	Downregulated in AD neocortex and temporal cortex [97,98]	Aβ homeostasis: *BACE1* [97,98]	miR-107 mimics rescued glutamate and Aβ toxicity in primary rodent and human neurons [92]	
miR-101	Downregulated in AD temporal cortex [99,100]	Aβ homeostasis: *BACE1, APP, Aβ42* [99,100,101,102]	miR-101 overexpression reduced Aβ load in rat hippocampal neurons [102]	Viral overexpression of miR-101 sponge in mouse hippocampus induced memory deficits [101]
miR-195	Downregulated in AD parietal cortex tissue [103]	Aβ homeostasis: *BACE1* [104] Lysosomal defects: *SYNJ1* [103]	Overexpression of miR-195 rescued lysosomal defects in iPSC-derived neurons from ADRD patients [103] and decreased Aβ plaque in N2a cells [104]	Viral overexpression in mouse models decreased Aβ plaque, tau hyperphosphorylation and rescued cognitive deficits in *ApoE4^+/+^* mice [103]
miR-34a	Upregulated in AD temporal cortex [88,105]	Aβ homeostasis: *ADAM10* [106] Tau homeostasis: *PTPA* [106] Synaptic plasticity: *VAMP, SYT1, HCN1, NR2A, GluR1, SIRT1* [105,106]	miR-34a mimics exacerbated, whereas miR-34a inhibitor protected against glutamate and Aβ toxicity in primary rodent and human neurons [92]	Overexpression of miR-34a induces rapid cognitive impairment in mouse model [106]
miR-26b	Upregulated in AD temporal cortex [107]	Tau homeostasis: *CDK5* [107] Aberrant cell cycle entry: *RB1* [107]	miR-26b inhibition protects mouse and human primary neurons against peroxide and Aβ toxicity [92,107]	
miR-203	Upregulated in FTD frontal cortex [108]	Genes in the neurodegeneration-associated synaptic (NAS) module: *BCL2L2, DGKB, MAPK10, VSNL1* [108]	Overexpressing mi-203 in mouse primary cortical neurons increased apoptosis [108]	Upregulation of miR-203 and corresponding downregulation of predicted targets in the cortex of TPR50 tau mice [108]
piRNAs	Various piRNA dysregulated in AD prefrontal cortex [109,110]			
**Linear lncRNAs**				
BACE1-AS (β Secretase Antisense Transcript)	Upregulated in AD cerebellum, hippocampus, cortex, and serum [111,112,113]	Aβ homeostasis: *BACE1, Aβ42* [111,112,113,114]	Knockdown *BACE1-AS* in SH-SY5Y cells reduced Aβ toxicity [113]	Hippocampal injection of *BACE1-AS* inhibitors reduced neuronal death in APP/PS1 mice [113] Activation of NRF2 by sulforaphane inhibited *BACE1* and *BACE1-AS* transcription and rescued cognitive deficits in 5xFAD mice [114]
NEAT1 (Nuclear Enriched Abundant Transcript 1)	Upregulated in AD temporal cortex and hippocampus [89,115,116]	Autophagy: *NEDD4L, PINK1* [117] Synaptic plasticity: promotes H3K9 dimethylation of *c-Fos* promoter to suppress c-Fos expression [118]	Overexpression of *NEAT1* exacerbated peroxide injury in N2A cells [119]	Hippocampal knockdown of *NEAT1* with siRNA improved memory in aged mice and vice versa [118] Viral knockdown of *NEAT1* rescued memory deficit in APP/PS1 mice [117]
17A	Upregulated in AD cortex [120]	Neurogenesis: nestin [121] Autophagy: *LC3B* [121]	Overexpression of *17A* in SH-SY5Y cells increased Aβ secretion [120] Downregulation of 17A in SH-SY5Y cells decreased apoptosis and Aβ42 secretion [121]	
BC200	Upregulated in AD cortex and hippocampus [122]	Aβ homeostasis: *APP* [123]		Hippocampal overexpression of rodent homolog *BC1* increased APP and Aβ expression and impaired memory, whereas inhibiting *BC1* was neuroprotective [123]
Enhancer RNAs	Various enhancer RNAs dysregulated in AD prefrontal cortex [124]			
**circRNAs**				
CDR1-AS (Cerebellar Degeneration-Related Protein 1 Antisense Transcript)	Downregulated in AD temporal cortex and hippocampus [125,126] Upregulated in AD parietal cortex [127]	Aβ homeostasis: *BACE1, APP* [128] Neuronal activity: miR-7 sponge, *c-Fos*, *EGR1, ARC* [129,130]	*CDR1-AS* overexpression promoted lysosomal degradation of *BACE1* and APP proteins in HEK293 and SH-SY5Y cells [128] *CDR1-AS* KO neurons showed dysfunctional synaptic neurotransmission [130]	*Cdr1-as* KO mice showed impaired sensorimotor gating, but normal memory acquisition [130]
circHOMER1 (circ-Homer Protein Homolog 1)	Downregulated in AD parietal [127] and frontal cortex [131], significantly associated with Braak score			
circKCNN2 (circ-Potassium Calcium-Activated Channel Subfamily N Member 2)	Downregulated in AD parietal [127] and frontal cortex [131], significantly associated with Braak score			
circDOCK1 (circ-Dedicator Of Cytokinesis 1)	Upregulated in AD parietal [127] and frontal cortex [131], significantly associated with Braak score			

Target gene abbreviations: ADAM10: ADAM Metallopeptidase Domain 10; APP: Amyloid β Precursor Protein; ARC: Activity Regulated Cytoskeleton Associated Protein; BACE1: β-Secretase 1; BCL2L2: BCL2-Like 2; BDNF: Brain Derived Neurotrophic Factor; CAPN2: Calpain 2; CDK5: Cyclin-Dependent Kinase 5; DGKB: Diacylglycerol Kinase β; EGR1: Early Growth Response 1; ERK1/2: Extracellular Signal-Regulated Kinase 1/2; EP300: E1A Binding Protein P300; FOXO3a: ForkheADRD Box O3a; GluR1: Glutamate Ionotropic Receptor AMPA Type Subunit 1; GSK-3β: Glycogen Synthase Kinase 3 β; HCN1: Hyperpolarization-Activated Cyclic Nucleotide Gated Potassium Channel 1; ITPKB: Inositol-Trisphosphate 3-Kinase B; LC3B: microtubule-associated proteins 1A/1B light chain 3B; MAPK10: Mitogen-Activated Protein Kinase 10; MAPT: Microtubule Associated Protein Tau; MeCP2: Methyl-CpG Binding Protein 2; NEDD4L: NEDD4-Like E3 Ubiquitin Protein Ligase; NR2A: NMDA receptor subunit 2A; NRF2: Nuclear Factor Erythroid 2-related Factor 2; p250GAP: p250 GTPase-activating protein; PINK1: PTEN Induced Kinase 1; PP2B: Protein Phosphatase-2B; PTPA: Protein Phosphatase 2 Phosphatase Activator; PTEN: Phosphatase And Tensin Homolog; RB1: Retinoblastoma-associated protein; RBFOX1: RNA Binding Protein Fox-1 Homolog 1; SIRT1: NADRD-dependent Deacetylase Sirtuin-1; SYNJ1: Synaptojanin 1; SYT1: Synaptotagmin 1; VAMP: Vesicle-Associated Membrane Protein 1; VSNL1: Visinin-Like 1.

## Data Availability

Not applicable.

## Abbreviations

AD	Alzheimer’s disease
ADRD	Alzheimer’s disease and related dementias
ALIS	Automated ligand identification system
APP	Amyloid–β precursor protein
AS	Antisense transcript
ASO	Antisense oligonucleotide
BACE1-AS	β-secretase 1 antisense transcript
BBB	Blood–brain barrier
BDNF	Brain-derived neurotropic factor
Cat-ELCCA	Catalytic enzyme-linked click chemistry assay
CDR1-AS	Cerebellar degeneration-related protein 1 antisense RNA
Chem-CLIP-Map-Seq	Chemical Cross-Linking and Isolation by Pull-down to Map Small Molecule-RNA Binding Sites
ChIP-seq	Chromatin immunoprecipitation-RNA-seq
circRNA	Circular RNA
CREB	cAMP response element-binding protein
DGCR8	DiGeorge critical region 8
DRUG-seq	Digital RNA with perturbation of Genes
ENCODE	Encyclopedia of DNA Elements
Exp5	Exportin 5
FANTOM	Functional Annotation of the Mammalian Genome
FDA	Food and Drug Administration
FID	Fluorescent indicator displacement
FP	Fluorescence polarization
FRET	Fluorescence resonance energy transfer
FTD	Frontotemporal dementia
HD	Huntington’s disease
HOTAIR	*HOX* antisense intergenic RNA
HTS	High-throughput screen
HTT	Huntingtin
ITC	Isothermal titration calorimetry
LBD	Lewy body dementia
LC-MS	Liquid chromatography-mass spectrometry
lncRNA	Long non-coding RNA
MALAT1	Metastasis-associated lung adenocarcinoma transcript 1
MED	Multiple etiology dementias
miRNA	MicroRNA
MoA	Mechanism of action
mRNA	Messenger RNA
ncRNA	Non-coding RNA
NMR	Nuclear magnetic resonance
PAIN	Pan-assay interference
PDB	Protein data bank
PK/PD	Pharmacokinetics and pharmacodynamics
PRC2	Polycomb repressive complex 2
PSEN1	Presenilin 1
PSEN2	Presenilin 2
RBP	RNA binding protein
REST	RE1-silencing transcription factor
RISC	RNA-induced silencing complex
rRNA	Ribosomal RNA
shRNA	Short hairpin RNA
siRNA	Small interfering RNA
SMN2	Survival of motor neuron 2
sncRNA	Small ncRNA
snoRNA	Small nucleolar RNA
snRNA	Small nuclear RNA
SPR	Surface plasmon resonance
TORNADO-seq	Targeted organoid sequencing
TRBP	Transactivating response RNA-binding protein
tRNA	Transfer RNA
VCID	Vascular contributions to cognitive impairment and dementia

## References

[1] GBD 2016 Dementia Collaborators (2019). GBD 2016 Dementia Collaborators Global, regional, and national burden of alzheimer’s disease and other dementias, 1990–2016: A systematic analysis for the global burden of disease study. Lancet Neurol..

[2] Corriveau R.A., Koroshetz W.J., Gladman J.T., Jeon S., Babcock D., Bennett D.A., Carmichael S.T., Dickinson S.L.-J., Dickson D.W., Emr M. (2017). Alzheimer’s Disease–Related Dementias Summit 2016: National research priorities. Neurology.

[3] Liu K.Y., Howard R. (2021). Can we learn lessons from the fda’s approval of aducanumab?. Nat. Rev. Neurol.

[4] Warner K.D., Hajdin C.E., Weeks K.M. (2018). Principles for targeting RNA with drug-like small molecules. Nat. Rev. Drug Discov..

[5] Barry G. (2014). Integrating the roles of long and small non-coding RNA in brain function and disease. Mol. Psychiatry.

[6] Mercer T.R., Dinger M.E., Sunkin S.M., Mehler M.F., Mattick J.S. (2008). Specific expression of long noncoding RNAs in the mouse brain. Proc. Natl. Acad. Sci. USA.

[7] He M., Liu Y., Wang X., Zhang M.Q., Hannon G.J., Huang Z.J. (2012). Cell-type-based analysis of microrna profiles in the mouse brain. Neuron.

[8] Derrien T., Johnson R., Bussotti G., Tanzer A., Djebali S., Tilgner H., Guernec G., Merkel A., Gonzalez D., Lagarde J. (2012). The GENCODE v7 catalogue of human long non-coding RNAs: Analysis of their structure, evolution and expression. Genome Res..

[9] Barry G., Mattick J. (2012). The role of regulatory RNA in cognitive evolution. Trends Cogn. Sci..

[10] Salta E., De Strooper B. (2017). Noncoding RNAs in neurodegeneration. Nat. Rev. Neurosci..

[11] Winkle M., El-Daly S.M., Fabbri M., Calin G.A. (2021). Noncoding RNA therapeutics—Challenges and potential solutions. Nat. Rev. Drug Discov..

[12] Gökirmak T., Nikan M., Wiechmann S., Prakash T.P., Tanowitz M., Seth P.P. (2021). Overcoming the challenges of tissue delivery for oligonucleotide therapeutics. Trends Pharmacol. Sci..

[13] Deverman B.E., Ravina B.M., Bankiewicz K.S., Paul S.M., Sah D.W.Y. (2018). Gene therapy for neurological disorders: Progress and prospects. Nat. Rev. Drug Discov..

[14] Bennett C.F., Krainer A.R., Cleveland D.W. (2019). Antisense oligonucleotide therapies for neurodegenerative diseases. Annu. Rev. Neurosci..

[15] Watts J.K., Brown R.H., Khvorova A. (2019). Nucleic Acid therapeutics for neurological diseases. Neurotherapeutics.

[16] Lipinski C.A., Lombardo F., Dominy B.W., Feeney P.J. (2001). Experimental and computational approaches to estimate solubility and permeability in drug discovery and development settings. Adv. Drug Deliv. Rev..

[17] Ajmone-Cat M.A., Bernardo A., Greco A., Minghetti L. (2010). Non-Steroidal Anti-Inflammatory Drugs and Brain Inflammation: Effects on Microglial Functions. Pharmaceuticals.

[18] Monroig P.D.C., Chen L., Zhang S., Calin G.A. (2014). Small molecule compounds targeting miRNAs for cancer therapy. Adv. Drug Deliv. Rev..

[19] Falese J.P., Donlic A., Hargrove A.E. (2021). Targeting RNA with small molecules: From fundamental principles towards the clinic. Chem. Soc. Rev..

[20] Martin W.J., Grandi P., Marcia M. (2021). Screening strategies for identifying RNA- and ribonucleoprotein-targeted compounds. Trends Pharmacol. Sci..

[21] Donlic A., Zafferani M., Padroni G., Puri M., Hargrove A.E. (2020). Regulation of MALAT1 triple helix stability and in vitro degradation by diphenylfurans. Nucleic Acids Res..

[22] Abulwerdi F.A., Xu W., Ageeli A.A., Yonkunas M., Arun G., Nam H., Schneekloth J.S., Dayie T.K., Spector D., Baird N. (2019). Selective small-molecule targeting of a triple helix encoded by the long noncoding RNA, MALAT1. ACS Chem. Biol..

[23] Rizvi N.F., Maria J.J.P.S., Nahvi A., Klappenbach J., Klein D.J., Curran P.J., Richards M.P., Chamberlin C., Saradjian P., Burchard J. (2019). Targeting RNA with small molecules: Identification of selective, RNA-binding small molecules occupying drug-like chemical space. SLAS Discov. Adv. Life Sci. R&D.

[24] Velagapudi S.P., Cameron M.D., Haga C.L., Rosenberg L.H., Lafitte M., Duckett D.R., Phinney D., Disney M.D. (2016). Design of a small molecule against an oncogenic noncoding RNA. Proc. Natl. Acad. Sci. USA.

[25] Palacino J., Swalley S.E., Song C., Cheung A.K., Shu L., Zhang X., Van Hoosear M., Shin Y., Chin D.N., Keller C.G. (2015). Smn2 splice modulators enhance u1-pre-mrna association and rescue sma mice. Nat. Chem. Biol..

[26] Naryshkin N.A., Weetall M., Dakka A., Narasimhan J., Zhao X., Feng Z., Ling K.K.Y., Karp G.M., Qi H., Woll M.G. (2014). SMN2 splicing modifiers improve motor function and longevity in mice with spinal muscular atrophy. Science.

[27] Chen J.L., Zhang P., Abe M., Aikawa H., Zhang L., Frank A.J., Zembryski T., Hubbs C., Park H., Withka J. (2020). Design, optimization, and study of small molecules that target tau pre-mrna and affect splicing. J. Am. Chem. Soc..

[28] Sterling T., Irwin J.J. (2015). Zinc 15--ligand discovery for everyone. J. Chem. Inf. Model..

[29] Dandapani S., Rosse G., Southall N., Salvino J.M., Thomas C.J. (2012). Selecting, acquiring, and using small molecule libraries for high-throughput screening. Curr. Protoc. Chem. Biol..

[30] Meng F., Xi Y., Huang J., Ayers P.W. (2021). A curated diverse molecular database of blood-brain barrier permeability with chemical descriptors. Sci. Data.

[31] Dowden H., Munro J. (2019). Trends in clinical success rates and therapeutic focus. Nat. Rev. Drug Discov..

[32] Horvath P., Aulner N., Bickle M., Davies A.M., Del Nery E., Ebner D., Montoya M.C., Östling P., Pietiäinen V., Price L.S. (2016). Screening out irrelevant cell-based models of disease. Nat. Rev. Drug Discov..

[33] Dawson T.M., Golde T.E., Lagier-Tourenne C. (2018). Animal models of neurodegenerative diseases. Nat. Neurosci..

[34] Treiber T., Treiber N., Meister G. (2018). Regulation of microRNA biogenesis and its crosstalk with other cellular pathways. Nat. Rev. Mol. Cell Biol..

[35] Quinn J.J., Chang H.Y. (2016). Unique features of long non-coding RNA biogenesis and function. Nat. Rev. Genet..

[36] Moore J.E., Purcaro M.J., Pratt H.E., Epstein C.B., Shoresh N., Adrian J., Kawli T., Davis C.A., Dobin A., The ENCODE Project Consortium (2020). Expanded encyclopaedias of DNA elements in the human and mouse genomes. Nature.

[37] Abugessaisa I., Ramilowski J.A., Lizio M., Severin J., Hasegawa A., Harshbarger J., Kondo A., Noguchi S., Yip C.W., Ooi J.L.C. (2020). FANTOM enters 20th year: Expansion of transcriptomic atlases and functional annotation of non-coding RNAs. Nucleic Acids Res..

[38] De Rie D., Abugessaisa I., Alam T., Arner E., Arner P., Ashoor H., Åström G., Babina M., Bertin N., Burroughs A.M. (2017). An integrated expression atlas of miRNAs and their promoters in human and mouse. Nat. Biotechnol..

[39] Khaled H.G., Feng H., Hu X., Sun X., Zheng W., Li P.P., Rudnicki D.D., Ye W., Chen Y.-C., Southall N. (2021). A high-throughput screening to identify small molecules that suppress huntingtin promoter activity or activate huntingtin-antisense promoter activity. Sci. Rep..

[40] Gumireddy K., Young D.D., Xiong X., Hogenesch J.B., Huang Q., Deiters A. (2008). Small-molecule inhibitors of MicroRNA miR-21 Function. Angew. Chem..

[41] Naro Y., Thomas M., Stephens M.D., Connelly C., Deiters A. (2015). Aryl amide small-molecule inhibitors of microRNA miR-21 function. Bioorg. Med. Chem. Lett..

[42] Bose D., Jayaraj G., Suryawanshi H., Agarwala P., Pore S.K., Banerjee R., Maiti S. (2011). The tuberculosis drug streptomycin as a potential cancer therapeutic: Inhibition of miR-21 function by directly targeting its precursor. Angew. Chem. Int. Ed..

[43] Xiao Z., Li C.H., Chan S., Xu F., Feng L., Wang Y., Jiang J.-D., Sung J.J.Y., Cheng C.H., Chen Y. (2014). A Small-Molecule Modulator of the tumor-suppressor miR34a inhibits the growth of hepatocellular carcinoma. Cancer Res..

[44] Young D.D., Connelly C., Grohmann C., Deiters A. (2010). small molecule modifiers of MicroRNA miR-122 function for the treatment of hepatitis c virus infection and hepatocellular carcinoma. J. Am. Chem. Soc..

[45] Khan M.R., Wellinger R.J., Laurent B. (2021). Exploring the alternative splicing of long noncoding RNAs. Trends Genet..

[46] Meganck R.M., Liu J., Hale A.E., Simon K.E., Fanous M.M., Vincent H.A., Wilusz J.E., Moorman N.J., Marzluff W.F., Asokan A. (2021). Engineering highly efficient backsplicing and translation of synthetic circRNAs. Mol. Ther.-Nucleic Acids.

[47] Tong Z., Cui Q., Wang J., Zhou Y. (2019). Transmir v2.0: An updated transcription factor-microrna regulation database. Nucleic Acids Res..

[48] Karra D., Dahm R. (2010). Transfection techniques for neuronal cells. J. Neurosci..

[49] Heitman L., van Veldhoven J.P.D., Zweemer A., Ye K., Brussee J., Ijzerman A.P. (2008). False positives in a reporter gene assay: Identification and synthesis of substituted N-Pyridin-2-ylbenzamides as competitive inhibitors of firefly luciferase. J. Med. Chem..

[50] Moffat J.G., Vincent F., Lee J.A., Eder J., Prunotto M. (2017). Opportunities and challenges in phenotypic drug discovery: An industry perspective. Nat. Rev. Drug Discov..

[51] Connelly C.M., Abulwerdi F.A., Schneekloth J.S. (2016). Discovery of RNA binding small molecules using small molecule microarrays. Small Mol. Microarrays.

[52] Connelly C.M., Numata T., Boer R.E., Moon M.H., Sinniah R.S., Barchi J.J., Ferre-D’Amare A.R., Schneekloth J.S. (2019). Synthetic ligands for preq1 riboswitches provide structural and mechanistic insights into targeting RNA tertiary structure. Nat. Commun..

[53] Wicks S.L., Hargrove A.E. (2019). Fluorescent indicator displacement assays to identify and characterize small molecule interactions with RNA. Methods.

[54] Disney M.D., Winkelsas A.M., Velagapudi S.P., Southern M., Fallahi M., Childs-Disney J.L. (2016). Inforna 2.0: A platform for the sequence-based design of small molecules targeting structured RNAs. ACS Chem. Biol..

[55] Padroni G., Patwardhan N.N., Schapira M., Hargrove A.E. (2020). Systematic analysis of the interactions driving small molecule–RNA recognition. RSC Med. Chem..

[56] Maveyraud L., Mourey L. (2020). Protein X-ray crystallography and drug discovery. Molecules.

[57] Freisz S., Lang K., Micura R., Dumas P., Ennifar E. (2008). Binding of aminoglycoside antibiotics to the duplex form of the HIV-1 genomic RNA dimerization initiation site. Angew. Chem. Int. Ed..

[58] Ren A., Patel D.J. (2014). c-di-AMP binds the ydaO riboswitch in two pseudo-symmetry–related pockets. Nat. Chem. Biol..

[59] Shortridge M.D., Walker M.J., Pavelitz T., Chen Y., Yang W., Varani G. (2017). A macrocyclic peptide ligand binds the oncogenic MicroRNA-21 precursor and suppresses dicer processing. ACS Chem. Biol..

[60] Velagapudi S.P., Li Y., Disney M.D. (2019). A cross-linking approach to map small molecule-RNA binding sites in cells. Bioorg. Med. Chem. Lett..

[61] Costales M.G., Aikawa H., Li Y., Childs-Disney J.L., Abegg D., Hoch D.G., Velagapudi S.P., Nakai Y., Khan T., Wang K.W. (2020). Small-molecule targeted recruitment of a nuclease to cleave an oncogenic RNA in a mouse model of metastatic cancer. Proc. Natl. Acad. Sci. USA.

[62] Meyer S.M., Williams C.C., Akahori Y., Tanaka T., Aikawa H., Tong Y., Childs-Disney J.L., Disney M.D. (2020). Small molecule recognition of disease-relevant RNA structures. Chem. Soc. Rev..

[63] Palazzo A.F., Lee E.S. (2015). Non-coding RNA: What is functional and what is junk?. Front. Genet..

[64] Wu P. (2020). Inhibition of RNA-binding proteins with small molecules. Nat. Rev. Chem..

[65] Roos M., Pradère U., Ngondo R.P., Behera A., Allegrini S., Civenni G., Zagalak J.A., Marchand J.-R., Menzi M., Towbin H. (2016). A small-molecule inhibitor of Lin28. ACS Chem. Biol..

[66] Wang L., Rowe R.G., Jaimes A., Yu C., Nam Y., Pearson D.S., Zhang J., Xie X., Marion W., Heffron G.J. (2018). Small-molecule inhibitors disrupt let-7 Oligouridylation and release the selective blockade of let-7 processing by LIN28. Cell Rep..

[67] Lorenz D.A., Kaur T., Kerk S.A., Gallagher E.E., Sandoval J., Garner A.L. (2018). Expansion of cat-ELCCA for the discovery of small molecule inhibitors of the Pre-let-7–Lin28 RNA–protein interaction. ACS Med. Chem. Lett..

[68] Watashi K., Yeung M.L., Starost M.F., Hosmane R.S., Jeang K.-T. (2010). Identification of small molecules that suppress MicroRNA function and reverse tumorigenesis. J. Biol. Chem..

[69] Masciarelli S., Quaranta R., Iosue I., Colotti G., Padula F., Varchi G., Fazi F., Del Rio A. (2014). A small-molecule targeting the microrna binding domain of argonaute 2 improves the retinoic acid differentiation response of the acute promyelocytic leukemia cell line NB4. ACS Chem. Biol..

[70] Uszczynska-Ratajczak B., Lagarde J., Frankish A., Guigó R., Johnson R. (2018). Towards a complete map of the human long non-coding RNA transcriptome. Nat. Rev. Genet..

[71] Hrdlickova R., Toloue M., Tian B. (2016). RNA-Seq methods for transcriptome analysis. Wiley Interdiscip. Rev. RNA.

[72] Isakova A., Fehlmann T., Keller A., Quake S.R. (2020). A mouse tissue atlas of small noncoding RNA. Proc. Natl. Acad. Sci. USA.

[73] Ye C., Ho D.J., Neri M., Yang C., Kulkarni T., Randhawa R., Henault M., Mostacci N., Farmer P., Renner S. (2018). DRUG-seq for miniaturized high-throughput transcriptome profiling in drug discovery. Nat. Commun..

[74] Norkin M., Ordóñez-Morán P., Huelsken J. (2021). High-content, targeted RNA-seq screening in organoids for drug discovery in colorectal cancer. Cell Rep..

[75] Nguyen L.D., Ehrlich B.E. (2020). Cellular mechanisms and treatments for chemobrain: Insight from aging and neurodegenerative diseases. EMBO Mol. Med..

[76] Verbist B., Klambauer G., Vervoort L., Talloen W., Shkedy Z., Thas O., Bender A., Göhlmann H.W., Hochreiter S. (2015). Using transcriptomics to guide lead optimization in drug discovery projects: Lessons learned from the QSTAR project. Drug Discov. Today.

[77] Baell J.B., Nissink J.W.M. (2017). Seven year itch: Pan-assay interference compounds (PAINS) in 2017—Utility and limitations. ACS Chem. Biol..

[78] Yang Z.-Y., Dong J., Yang Z.-J., Lu A.-P., Hou T.-J., Cao D.-S. (2020). Structural analysis and identification of false positive hits in luciferase-based assays. J. Chem. Inf. Model..

[79] Nance E., Pun S.H., Saigal R., Sellers D.L. (2021). Drug delivery to the central nervous system. Nat. Rev. Mater..

[80] Benek O., Korabecny J., Soukup O. (2020). A perspective on multi-target drugs for alzheimer’s disease. Trends Pharmacol. Sci..

[81] Gashaw I., Ellinghaus P., Sommer A., Asadullah K. (2011). What makes a good drug target?. Drug Discov Today.

[82] Wong H.-K., Veremeyko T., Patel N., Lemere C.A., Walsh D.M., Esau C., Vanderburg C., Krichevsky A.M. (2013). De-repression of FOXO3a death axis by microRNA-132 and -212 causes neuronal apoptosis in Alzheimer’s disease. Hum. Mol. Genet..

[83] Lau P., Bossers K., Janky R., Salta E., Frigerio C.S., Barbash S., Rothman R., Sierksma A.S., Thathiah A., Greenberg D. (2013). Alteration of the microrna network during the progression of alzheimer’s disease. EMBO Mol. Med..

[84] Patrick E., Rajagopal S., Wong H.-K.A., McCabe C., Xu J., Tang A., Imboywa S.H., Schneider J.A., Pochet N., Krichevsky A.M. (2017). Dissecting the role of non-coding RNAs in the accumulation of amyloid and tau neuropathologies in Alzheimer’s disease. Mol. Neurodegener..

[85] Pichler S., Gu W., Hartl D., Gasparoni G., Leidinger P., Keller A., Meese E., Mayhaus M., Hampel H., Riemenschneider M. (2017). The mirnome of alzheimer’s disease: Consistent downregulation of the mir-132/212 cluster. Neurobiol. Aging.

[86] Cogswell J.P., Ward J., Taylor I.A., Waters M., Shi Y., Cannon B., Kelnar K., Kemppainen J., Brown D., Chen C. (2008). Identification of miRNA changes in alzheimer’s disease brain and CSF yields putative biomarkers and insights into disease pathways. J. Alzheimer’s Dis..

[87] Salta E., Sierksma A., Eynden E.V., De Strooper B. (2016). miR-132 loss de-represses ITPKB and aggravates amyloid and TAU pathology in Alzheimer’s brain. EMBO Mol. Med..

[88] Li Q.S., Cai D. (2021). Integrated mirna-seq and mrna-seq study to identify mirnas associated with alzheimer’s disease using post-mortem brain tissue samples. Front. Neurosci..

[89] Annese A., Manzari C., Lionetti C., Picardi E., Horner D.S., Chiara M., Caratozzolo M.F., Tullo A., Fosso B., Pesole G. (2018). Whole transcriptome profiling of Late-Onset Alzheimer’s Disease patients provides insights into the molecular changes involved in the disease. Sci. Rep..

[90] Hebert S.S., Wang W.X., Zhu Q., Nelson P.T. (2013). A study of small rnas from cerebral neocortex of pathology-verified alzheimer’s disease, dementia with lewy bodies, hippocampal sclerosis, frontotemporal lobar dementia, and non-demented human controls. J. Alzheimers Dis..

[91] Chen-Plotkin A.S., Unger T.L., Gallagher M.D., Bill E., Kwong L.K., Volpicelli-Daley L., Busch J.I., Akle S., Grossman M., Van Deerlin V. (2012). Tmem106b, the risk gene for frontotemporal dementia, is regulated by the microrna-132/212 cluster and affects progranulin pathways. J. Neurosci..

[92] El Fatimy R., Li S., Chen Z., Mushannen T., Gongala S., Wei Z., Balu D., Rabinovsky R., Cantlon A., Elkhal A. (2018). MicroRNA-132 provides neuroprotection for tauopathies via multiple signaling pathways. Acta Neuropathol..

[93] Smith P.Y., Hernandez-Rapp J., Jolivette F., Lecours C., Bisht K., Goupil C., Dorval V., Parsi S., Morin F., Planel E. (2015). Mir-132/212 deficiency impairs tau metabolism and promotes pathological aggregation in vivo. Hum. Mol. Genet..

[94] Hernandez-Rapp J., Smith P.Y., Filali M., Goupil C., Planel E., Magill S.T., Goodman R.H., Hebert S.S. (2015). Memory formation and retention are affected in adult mir-132/212 knockout mice. Behav. Brain Res..

[95] Vo N., Klein M.E., Varlamova O., Keller D.M., Yamamoto T., Goodman R.H., Impey S. (2005). A camp-response element binding protein-induced microrna regulates neuronal morphogenesis. Proc. Natl. Acad. Sci. USA.

[96] Walgrave H., Balusu S., Snoeck S., Eynden E.V., Craessaerts K., Thrupp N., Wolfs L., Horré K., Fourne Y., Ronisz A. (2021). Restoring miR-132 expression rescues adult hippocampal neurogenesis and memory deficits in Alzheimer’s disease. Cell Stem Cell.

[97] Nelson P.T., Wang W.-X. (2010). MiR-107 is reduced in alzheimer’s disease brain neocortex: Validation study. J. Alzheimer’s Dis..

[98] Wang W.X., Rajeev B.W., Stromberg A.J., Ren N., Tang G., Huang Q., Rigoutsos I., Nelson P.T. (2008). The expression of microrna mir-107 decreases early in alzheimer’s disease and may accelerate disease progression through regulation of β-site amyloid precursor protein-cleaving enzyme. J. Neurosci..

[99] Hebert S.S., Horre K., Nicolai L., Papadopoulou A.S., Mandemakers W., Silahtaroglu A.N., Kauppinen S., Delacourte A., De Strooper B. (2008). Loss of microrna cluster mir-29a/b-1 in sporadic alzheimer’s disease correlates with increased bace1/β-secretase expression. Proc. Natl. Acad. Sci. USA.

[100] Nunez-Iglesias J., Liu C.-C., Morgan T.E., Finch C.E., Zhou X.J. (2010). Joint genome-wide profiling of miRNA and mRNA expression in alzheimer’s disease cortex reveals altered miRNA regulation. PLoS ONE.

[101] Barbato C., Giacovazzo G., Albiero F., Scardigli R., Scopa C., Ciotti M.T., Strimpakos G., Coccurello R., Ruberti F. (2020). Cognitive decline and modulation of alzheimer’s disease-related genes after inhibition of MicroRNA-101 in Mouse hippocampal neurons. Mol. Neurobiol..

[102] Vilardo E., Barbato C., Ciotti M., Cogoni C., Ruberti F. (2010). MicroRNA-101 regulates amyloid precursor protein expression in hippocampal neurons. J. Biol. Chem..

[103] Cao J., Huang M., Guo L., Zhu L., Hou J., Zhang L., Pero A., Ng S., El Gaamouch F., Elder G. (2020). MicroRNA-195 rescues ApoE4-induced cognitive deficits and lysosomal defects in Alzheimer’s disease pathogenesis. Mol. Psychiatry.

[104] Zhu H.-C., Wang L.-M., Wang M., Song B., Tan S., Teng J.-F., Duan D.-X. (2012). MicroRNA-195 downregulates Alzheimer’s disease amyloid-β production by targeting BACE1. Brain Res. Bull..

[105] Sarkar S., Jun S., Rellick S., Quintana D.D., Cavendish J.Z., Simpkins J.W. (2016). Expression of microrna-34a in alzheimer’s disease brain targets genes linked to synaptic plasticity, energy metabolism, and resting state network activity. Brain Res..

[106] Sarkar S., Engler-Chiurazzi E., Cavendish J., Povroznik J., Russell A., Quintana D., Mathers P., Simpkins J. (2019). Over-expression of miR-34a induces rapid cognitive impairment and Alzheimer’s disease-like pathology. Brain Res..

[107] Absalon S., Kochanek D.M., Raghavan V., Krichevsky A.M. (2013). MiR-26b, Upregulated in Alzheimer’s Disease, activates cell cycle entry, tau-phosphorylation, and apoptosis in postmitotic neurons. J. Neurosci..

[108] Swarup V., Hinz F.I., Rexach J.E., Noguchi K.-I., Toyoshiba H., Oda A., Hirai K., Sarkar A., Seyfried N.T., Chen C. (2018). Identification of evolutionarily conserved gene networks mediating neurodegenerative dementia. Nat. Med..

[109] Jain G., Stuendl A., Rao P., Berulava T., Pena Centeno T., Kaurani L., Burkhardt S., Delalle I., Kornhuber J., Hull M. (2019). A combined mirna-pirna signature to detect alzheimer’s disease. Transl. Psychiatry.

[110] Qiu W., Guo X., Lin X., Yang Q., Zhang W., Zhang Y., Zuo L., Zhu Y., Li C.-S.R., Ma C. (2017). Transcriptome-wide piRNA profiling in human brains of Alzheimer’s disease. Neurobiol. Aging.

[111] Faghihi M.A., Modarresi F., Khalil A.M., Wood D.E., Sahagan B.G., Morgan T.E., Finch C.E., St Laurent G., Kenny P.J., Wahlestedt C. (2008). Expression of a noncoding RNA is elevated in alzheimer’s disease and drives rapid feed-forward regulation of β-secretase. Nat. Med..

[112] Faghihi M.A., Zhang M., Huang J., Modarresi F., Van der Brug M.P., Nalls M.A., Cookson M.R., St-Laurent G., Wahlestedt C. (2010). Evidence for natural antisense transcript-mediated inhibition of microrna function. Genome Biol..

[113] Zhou Y., Ge Y., Liu Q., Li Y.-X., Chao X., Guan J.-J., Diwu Y.-C., Zhang Q. (2020). LncRNA BACE1-AS Promotes Autophagy-Mediated Neuronal Damage Through The miR-214-3p/ATG5 Signalling Axis In Alzheimer’s Disease. Neuroscience.

[114] Bahn G., Park J.S., Yun U.J., Lee Y.J., Choi Y., Park J.S., Baek S.H., Choi B.Y., Cho Y.S., Kim H.K. (2019). Nrf2/are pathway negatively regulates bace1 expression and ameliorates cognitive deficits in mouse alzheimer’s models. Proc. Natl. Acad. Sci. USA.

[115] Spreafico M., Grillo B., Rusconi F., Battaglioli E., Venturin M. (2018). Multiple layers of cdk5r1 regulation in alzheimer’s disease implicate long non-coding RNAs. Int. J. Mol. Sci..

[116] Puthiyedth N., Riveros C., Berretta R., Moscato P. (2016). Identification of differentially expressed genes through integrated study of alzheimer’s disease affected brain regions. PLoS ONE.

[117] Huang Z., Zhao J., Wang W., Zhou J., Zhang J. (2020). Depletion of LncRNA NEAT1 rescues mitochondrial dysfunction through NEDD4L-Dependent PINK1 degradation in animal models of alzheimer’s disease. Front. Cell. Neurosci..

[118] Butler A.A., Johnston D.R., Kaur S., Lubin F.D. (2019). Long noncoding RNA NEAT1 mediates neuronal histone methylation and age-related memory impairment. Sci. Signal..

[119] Sunwoo J.-S., Lee S.-T., Im W., Lee M., Byun J.-I., Jung K.-H., Park K.-I., Jung K.-Y., Lee S.K., Chu K. (2016). Altered expression of the long noncoding RNA NEAT1 in huntington’s disease. Mol. Neurobiol..

[120] Massone S., Vassallo I., Fiorino G., Castelnuovo M., Barbieri F., Borghi R., Tabaton M., Robello M., Gatta E., Russo C. (2011). 17A, a novel non-coding RNA, regulates GABA B alternative splicing and signaling in response to inflammatory stimuli and in Alzheimer disease. Neurobiol. Dis..

[121] Wang X., Zhang M., Liu H. (2019). LncRNA17A regulates autophagy and apoptosis of SH-SY5Y cell line as an in vitro model for Alzheimer’s disease. Biosci. Biotechnol. Biochem..

[122] Mus E., Hof P.R., Tiedge H. (2007). Dendritic BC200 RNA in aging and in Alzheimer’s disease. Proc. Natl. Acad. Sci. USA.

[123] Zhang T., Pang P., Fang Z., Guo Y., Li H., Li X., Tian T., Yang X., Chen W., Shu S. (2017). Expression of BC1 impairs spatial learning and memory in alzheimer’s disease via APP translation. Mol. Neurobiol..

[124] Li P., Marshall L., Oh G., Jakubowski J.L., Groot D., He Y., Wang T., Petronis A., Labrie V. (2019). Epigenetic dysregulation of enhancers in neurons is associated with Alzheimer’s disease pathology and cognitive symptoms. Nat. Commun..

[125] Lukiw W.J. (2013). Circular RNA (circRNA) in Alzheimer’s disease (AD). Front. Genet..

[126] Zhao Y., Alexandrov P.N., Jaber V., Lukiw W.J. (2016). Deficiency in the Ubiquitin Conjugating Enzyme UBE2A in alzheimer’s disease (AD) is linked to deficits in a natural circular miRNA-7 sponge (circRNA; ciRS-7). Genes.

[127] Dube U., Del-Aguila J.L., Li Z., Budde J., Jiang S., Hsu S., Ibanez L., Fernandez M.V., Farias F., Norton J. (2019). An atlas of cortical circular RNA expression in Alzheimer disease brains demonstrates clinical and pathological associations. Nat. Neurosci..

[128] Shi Z., Chen T., Yao Q., Zheng L., Zhang Z., Wang J., Hu Z., Cui H., Han Y., Han X. (2017). The circular RNA cirs-7 promotes app and bace1 degradation in an nf-kappab-dependent manner. FEBS J..

[129] Hansen T.B., Jensen T.I., Clausen B.H., Bramsen J.B., Finsen B., Damgaard C.K., Kjems J. (2013). Natural RNA circles function as efficient microRNA sponges. Nature.

[130] Piwecka M., Glažar P., Hernandez-Miranda L.R., Memczak S., Wolf S.A., Rybak-Wolf A., Filipchyk A., Klironomos F., Cerda Jara C.A., Fenske P. (2017). Loss of a mammalian circular RNA locus causes miRNA deregulation and affects brain function. Science.

[131] Cervera-Carles L., Dols-Icardo O., Molina-Porcel L., Alcolea D., Cervantes-Gonzalez A., Muñoz L., Clarimon J. (2020). Assessing circular RNAs in Alzheimer’s disease and frontotemporal lobar degeneration. Neurobiol. Aging.

[132] Costales M.G., Haga C.L., Velagapudi S.P., Childs-Disney J.L., Phinney D.G., Disney M.D. (2017). Small Molecule Inhibition of microRNA-210 Reprograms an Oncogenic Hypoxic Circuit. J. Am. Chem. Soc..

[133] Salta E., De Strooper B. (2016). microRNA-132: A key noncoding RNA operating in the cellular phase of Alzheimer’s disease. FASEB J..

[134] He A.T., Liu J., Li F., Yang B.B. (2021). Targeting circular RNAs as a therapeutic approach: Current strategies and challenges. Signal. Transduct. Target. Ther..

[135] Long J.M., Holtzman D.M. (2019). Alzheimer disease: An update on pathobiology and treatment strategies. Cell.

[136] Jansen I.E., Savage J.E., Watanabe K., Bryois J., Williams D., Steinberg S., Sealock J., Karlsson I., Hägg S., Athanasiu L. (2019). Genome-wide meta-analysis identifies new loci and functional pathways influencing Alzheimer’s disease risk. Nat. Genet..

